# The determinants and impact of diagnostic delay in lymphoma in a TB and HIV endemic setting

**DOI:** 10.1186/s12885-019-5586-4

**Published:** 2019-04-25

**Authors:** Katherine Antel, Carly Levetan, Zainab Mohamed, Vernon J Louw, Jenna Oosthuizen, Gary Maartens, Estelle Verburgh

**Affiliations:** 1 Division of Haematology, Department of Internal Medicine, University of Cape Town, Cape Town, South Africa; 2 Medical advisor, Cell and Gene Therapy, Novartis Oncology, Sydney, Australia; 3 Department of Oncology, University of Cape Town, Cape Town, South Africa; 4 Division of Pharmacology, Department of Internal Medicine, University of Cape Town, Cape Town, South Africa

**Keywords:** Lymphoma, Diagnosis, Tuberculosis, HIV, FNAC (fine-needle aspiration)

## Abstract

**Background:**

Little is known about the pathway to diagnosis of lymphoma in Sub-Saharan Africa, despite the increased risk of lymphoma in people living with HIV (PLHIV). The challenges of diagnosis in this setting include diagnostic confusion with extrapulmonary tuberculosis (EPTB), which commonly causes lymphadenopathy in PLHIV.

**Methods:**

We analysed the time to diagnosis and treatment in patients using predetermined time intervals. Univariate and multivariable analyses were performed to determine the relationship between patient and disease-specific variables with delays to diagnosis. We were particularly interested in the impact of HIV, empiric tuberculosis therapy and fine-needle aspirate for cytology (FNAC) in contributing to delay.

**Results:**

Patients (*n* = 163), 29% HIV-infected, waited a median of 4 weeks before seeking medical attention. It took a median of 7 weeks for the diagnosis of lymphoma to be made from the time the patient sought medical attention, termed the *healthcare practitioner interval*. In multivariable logistic regression analysis, diagnostic delay > 6 weeks was associated with late-stage disease (OR 2.3, 95% CI 1.1–5.2) and Hodgkin lymphoma (HL) (OR 3.0, 95% CI 1.1–8.0). HIV status was not associated with diagnostic delay (OR 0.9, 95% CI 0.3–2.2). The median time to diagnosis was a median of 4 weeks longer for patients on tuberculous (TB) therapy (*n* = 16, *p* = 0.28) and patients who underwent an FNAC (*n* = 63, *p* = 0.04). Where FNAC was performed, it was diagnostic for lymphoma in only 11%. Diagnostic delay was not associated with overall survival.

**Conclusions:**

Time-to-diagnosis of lymphoma in South Africa was similar to that reported from high-income countries and shows significant periods of delay between the onset of symptoms to diagnosis and treatment. The longest period of delay was in the *health practitioner interval*. Education regarding the significance of lymphadenopathy for both patients and health care practitioners and appropriate investigative steps preferably by best-practice algorithms specific to TB-endemic areas are needed to shorten the time-to-diagnosis of lymphoma.

**Electronic supplementary material:**

The online version of this article (10.1186/s12885-019-5586-4) contains supplementary material, which is available to authorized users.

## Background

Lymphoma has emerged as the leading cancer-cause for mortality in people living with HIV (PLHIV) [1–4] and despite antiretroviral therapy (ARVs) shows a markedly increased risk in PLHIV, with a standardised incidence ratio of 11.5 for Non-Hodgkin lymphoma (NHL) and 7.7 for Hodgkin lymphoma (HL) [4]. The diagnosis of lymphoma in Sub-Saharan Africa (SSA) is complicated by overlapping symptoms due to extra-pulmonary tuberculosis (EPTB), and difficulties in accessing lymph node biopsies in poor resource settings. There is a paucity of literature on the time-to-diagnosis, and on the impact of the unique barriers to the diagnosis of lymphoma in SSA.

EPTB accounts for 20–70% of TB cases in PLHIV [5, 6] with lymph nodes and pleura the most common extrapulmonary sites of involvement [6, 7]. Despite recent advances in rapid molecular TB diagnostic techniques, EPTB remains challenging to diagnose and in TB endemic areas, PLHIV presenting with lymphadenopathy are commonly placed on empiric TB therapy. The fine-needle aspirate for cytology (FNAC) is typically the first investigation performed by the physician but has both poor sensitivity for lymphoma [8] and TB (acid-fast bacilli are seen in roughly 30% of cases) [9]. Furthermore, the FNAC may show poorly-formed granulomas in both, widely misinterpreted as specific for TB. The combination of overlapping symptoms (constitutional symptoms, lymphadenopathy, cytopenias and pleural effusion), and inadequacy of FNAC, puts patients in TB endemic areas at a unique risk for delayed diagnosis of lymphoma. This is illustrated by African studies reporting that 25–85% of patients are on TB therapy (most often for a presumed diagnosis of EPTB) at the time of diagnosis of lymphoma [10, 11].

Challenges in the diagnosis of lymphoma in non-TB endemic areas have been highlighted in previous studies, with lymphoma described as having the second longest time from symptom onset to diagnosis of any malignancy [12]. Numerous barriers to diagnosis of lymphoma have been identified, such as the insidious onset of symptoms and lack of specificity for diagnosis [13], lack of a distinct referral pathway for lymphadenopathy, the poor contribution of the FNAC to diagnosis and a need for adequate tissue sampling [12, 14, 15]. A model has been developed to assess time-to-diagnosis and ensure consistency between studies [16] which was applied in this study. Lymphoma diagnostic delay has not been previously examined in SSA.

In this study, we retrospectively review the pathway to diagnosis of aggressive NHL and HL at Groote Schuur Hospital, a large tertiary hospital in Cape Town, South Africa, which is at the epicentre of the TB and HIV pandemics [5]. Secondary aims were to describe the determinants of diagnostic delay, including TB therapy and FNAC as an initial sampling method, and the impact of diagnostic delay on overall survival.

## Methods

### Patient selection

Consecutive patients (age > 16) diagnosed with aggressive NHL as defined by Conners [17] and HL referred to Groote Schuur Hospital between June 2012 and June 2014 were identified from clinical and pathology databases (*n* = 163) and included in the study. Subtypes of NHL that were included were: diffuse-large B cell lymphoma (DLBCL), mantle cell lymphoma, primary mediastinal B cell lymphoma, plasmablastic lymphoma, T-cell/histiocyte rich large B-cell lymphoma, and T-cell lymphomas (anaplastic large cell, peripheral T cell NOS, angioimmunoblastic T cell, NK cell T-cell lymphoma). Indolent (follicular lymphoma, small lymphocytic lymphoma, chronic lymphocytic leukaemia) and acute leukaemia-like lymphomas (Burkitt’s and lymphoblastic lymphoma), and primary central nervous system (CNS) lymphoma were excluded because of their very different natural histories. Both HIV positive and negative patients were included. Due to the time period of the study, the 2008 WHO classification of lymphoma [18] was used to classify patients into lymphoma type.

### Data sources for clinical and pathology information

Demographic and baseline clinical characteristics, the type of lymphoma, biopsy dates, and bone marrow involvement were extracted by retrospective chart review and the National Health Laboratory Systems database. Lymphoma subtype was confirmed by pathology report. In order to evaluate the timing of onset and description of symptomatology one researcher conducted semi-structured interviews with 80 patients (via telephone or in person). In a further 83 patients, information regarding symptom onset and description was retrieved from patient records (these patients were either deceased or not contactable). Socio-economic status of the patients were ascertained from administrative records, which were self-reported income levels. Interviews were conducted by a health care professional (doctor) and conducted in the oncology clinic, according to a standard set of questions. In the case of Xhosa-speaking patients, a nurse was asked to translate (there was no trained translator available).

The time to diagnosis and treatment was described in the following time intervals: *patient interval* (self-reported symptom onset to first healthcare provider consultation), *healthcare practitioner interval* (first healthcare contact to a diagnostic biopsy), *referral interval* (histopathological diagnosis to referral to the specialist haematology/−oncology clinic) and *treatment interval* (specialist clinic visit to treatment start date). We defined diagnostic delay as > 6 weeks healthcare practitioner interval, as per Nikonova et al. which loosely corresponds to a longer period than that defined as acceptable in the UK, with the NICE guidelines targeting a specialist referral within 2 weeks for patients suspected of having lymphoma [19], and a further 2–3 weeks to biopsy [20]. Performance score was measured by the Eastern Cooperative Oncology Group (ECOG) score and assigned according to the doctor who interviewed the patient or abstracted from clinical records. Staging of lymphoma was by the Cotswolds-modified Ann Arbor classification, determined by CT scan and bone marrow aspiration and trephine biopsy (taken from patient records). Chemotherapy given within 3 days of referral was defined as ‘emergency chemotherapy’.

### Statistical analysis

Categorical and continuous variables were summarised with frequencies and percentages or medians and inter-quartile ranges respectively. Univariate comparisons between categorical variables were performed with the chi-squared test. Medians for non-parametric data were compared using the Wilcoxon Rank-Sum test, or Kruskal-Wallis test if more than one categorical variable. Multivariable logistic regression analysis was performed to calculate odds ratios (OR) with a 95% confidence interval (CI) to assess an association between clinically relevant covariates and diagnostic delay as defined above. Covariates in the multivariable logistic regression analysis were type of lymphoma (HL or NHL), HIV status, age (< or ≥ 50 years), stage of lymphoma (late-stage(III/IV) or early-stage (I/II)), an FNAC prior to diagnostic biopsy, and emergency chemotherapy.

A multivariable Cox proportional hazards model was developed to assess the impact of delays on overall survival (OS) and 2-year survival. Covariates in the Cox proportional hazards model were type of lymphoma, HIV status, age (< or ≥ 50 years), stage, performance status, emergency treatment and delay in diagnosis. Kaplan-Meier survival curves were constructed to assess 5-year OS at November 2017. Survival time was calculated from the date of diagnostic biopsy until date of death from any cause or date last seen alive. Date of death and date last seen were obtained through hospital record and laboratory systems. The statistical significance of the difference between survival distributions was determined by means of the log-rank test.

STATA v14.2 [21] was used for all descriptive and quantitative analyses. The study was approved by the university and hospital ethics review boards.

## Results

### Pathological characteristics

**Fig. 1:**
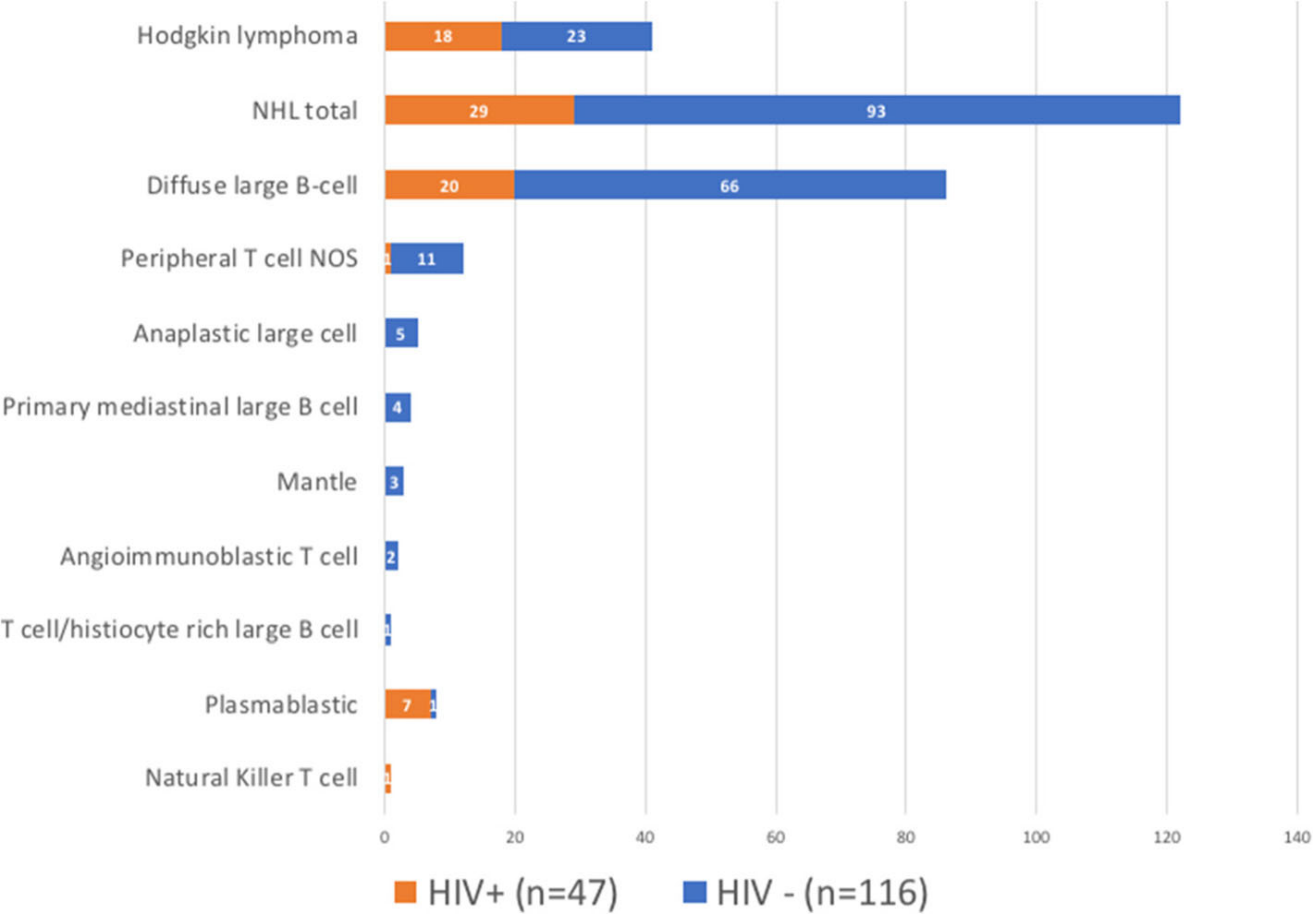
Type and subtype of lymphoma

Among 163 consecutive patients with newly diagnosed aggressive lymphoma between 2012 and 2014, 122 patients (75%) had NHL and 41 (25%) had HL. The histological distribution of NHL and HIV status is depicted in Fig. 1, diffuse large B-cell lymphoma (DBLCL) accounted for 70% of NHL. Histological diagnosis was made by a histopathologist and taken from patient results. Twenty-nine percent of patients were HIV-infected (*n* = 47): with a median CD4 count of 143 cells/mm^3^ (IQR, 69–274) and 61% on antiretroviral therapy at the time of lymphoma diagnosis.

Median age at diagnosis was 48 years (range 15–86 years) and 58% of patients were males.

Sixty-five percent of patients presented with late-stage disease (defined as stage 3 or 4), and in 25% of patients the bone marrow was involved at diagnosis. No patients had CNS involvement at diagnosis.

The majority of the study population were from a low socio-economic background, with 70% of patients on a social grant or having a monthly household income of less than R 4164 (£ 251). The characteristics of the population are summarised in Table 1.

**Table 1:** Sample characteristics and disease presenting features

	All (*n* = 163)		HIV - (*n* = 116)		HIV + (*n* = 47)		*P*-value	NHL (*n* = 122)		HL (*n* = 41)		*P*-value
Sex												
Male	94	58%	72	62%	22	47%	0.07	70	57%	24	59%	
Female	69	42%	44	38%	25	53%		52	43%	17	41%	0.90
Age												
Median (IQR)	48 (33–59)		54 (37–62)		38 (30–47)		< 0.01	51 (38–61)		35 (28–45)		< 0.01
< 18	3	2%	3	3%	0	0%		2	2%	1	2%	
18–39	57	35%	30	26%	27	57%		34	28%	23	56%	
40–59	66	40%	46	40%	20	43%		52	43%	14	34%	
60–79	35	21%	35	30%	0	0%		32	26%	3	7%	
80 or older	2	1%	2	2%	0	0%		2	2%	0	0%	
Race												
Mixed ancestry	81	50%	77	66%	4	9%	< 0.01	62	51%	19	46%	
Black	59	36%	18	16%	41	87%		40	33%	19	46%	
White	23	14%	21	18%	2	4%		20	16%	3	7%	0.18
Performance score												
ECOG 0	19	12%	14	12%	5	11%	0.97	11	9%	8	20%	
ECOG 1	74	45%	54	47%	20	43%		53	43%	21	51%	
ECOG 2	27	17%	19	16%	8	17%		21	17%	6	15%	
ECOG 3	32	20%	22	19%	10	21%		27	22%	5	12%	
ECOG 4	11	7%	7	6%	4	9%		10	8%	1	2%	0.17
Stage of disease at presentation												
Stage 1	23	14%	18	16%	5	11%	0.23	22	18%	1	2%	0.04
Stage 2	35	21%	27	23%	8	17%		28	23%	7	17%	
Stage 3	19	12%	10	9%	9	19%		13	11%	6	15%	
Stage 4	86	53%	61	53%	25	53%		59	48%	27	66%	
Bone marrow involved at diagnosis	41	25%	23	20%	18	38%	0.03	23	19%	18	44%	< 0.01
Clinical presentation and investigations												
Peripheral lymphadenopathy	104	64%	73	63%	31	66%	0.72	69	57%	35	85%	< 0.01
B Symptoms	96	59%	66	57%	30	64%	0.42	66	54%	30	73%	0.03
Diagnosis made on bone marrow	10	6%	5	4%	5	11%	0.66	4	3%	6	15%	0.23
On TB therapy at diagnosis	16	10%	4	3%	12	26%	< 0.01	5	4%	11	27%	< 0.01
FNAC												
Performed	63	39%	44	38%	19	40%	0.77	39	32%	24	59%	< 0.01
Not performed	100	61%	72	62%	28	60%		83	68%	17	41%	

### Presenting features leading to the diagnosis of lymphoma

The majority of patients had peripheral lymphadenopathy (64%) and B symptoms (59%) at diagnosis. In a small proportion of patients (6%) the diagnosis was made on biopsy of the bone marrow as opposed to a lymph node biopsy. In comparison with NHL, patients with HL were significantly more likely to present with peripheral lymphadenopathy (*p* < 0.01), have B symptoms (*p* = 0.03), have bone marrow involvement at diagnosis (*p* < 0.01) and have late-stage disease at diagnosis (*p* = 0.04) (Table 1).

Ten percent of patients were on TB therapy at diagnosis (*n* = 16, 10%), this was higher in the HL group than in NHL, with 27% on TB therapy (*n* = 2 proven TB, *n* = 14 empiric TB treatment). Patients with HL compared with NHL (OR 8.6, 95% CI 2.8–26.6) and patients with HIV (compared with not HIV-infected) were more likely to be on TB therapy at diagnosis (OR 9.6, 95% CI 2.9–31.7).

Sixteen percent (*n* = 25) of patients required emergency chemotherapy. Late stage disease (76% vs 60%, *p* = 0.14) and a poor performance score with an ECOG of 3 or 4 (40% vs 21%, *p* = 0.04) was more common in the emergency chemotherapy group. This group also had a shorter median health practitioner interval (4.3 weeks vs 8 weeks (IQR 3.0–17), *p* = 0.04).

### Utility of a fine needle aspiration in the diagnosis of aggressive lymphoma

A total of 90 FNACs were performed on 63 patients, 44% of patients had more than one FNAC with 2,3, and 4 FNACs performed in 16, 8 and 4 patients respectively. Of these 63 patients, 24 (38%) had HL and 39 (62%), NHL. The findings were suggestive (but not diagnostic) of lymphoma in only 10 of the 90 FNACs (11%), with the remaining FNAC results reported as unsatisfactory for assessment (*n* = 29), benign reactive changes (*n* = 26), atypical (*n* = 18), other malignancy (*n* = 3), and findings suggestive of TB (granulomas, acid-fast bacilli or caseous necrosis, *n* = 4). The time from the first FNAC to the diagnostic biopsy was a median of 23 days (IQR 12–67 days).

### Interval to diagnosis and treatment

**Fig. 2:**
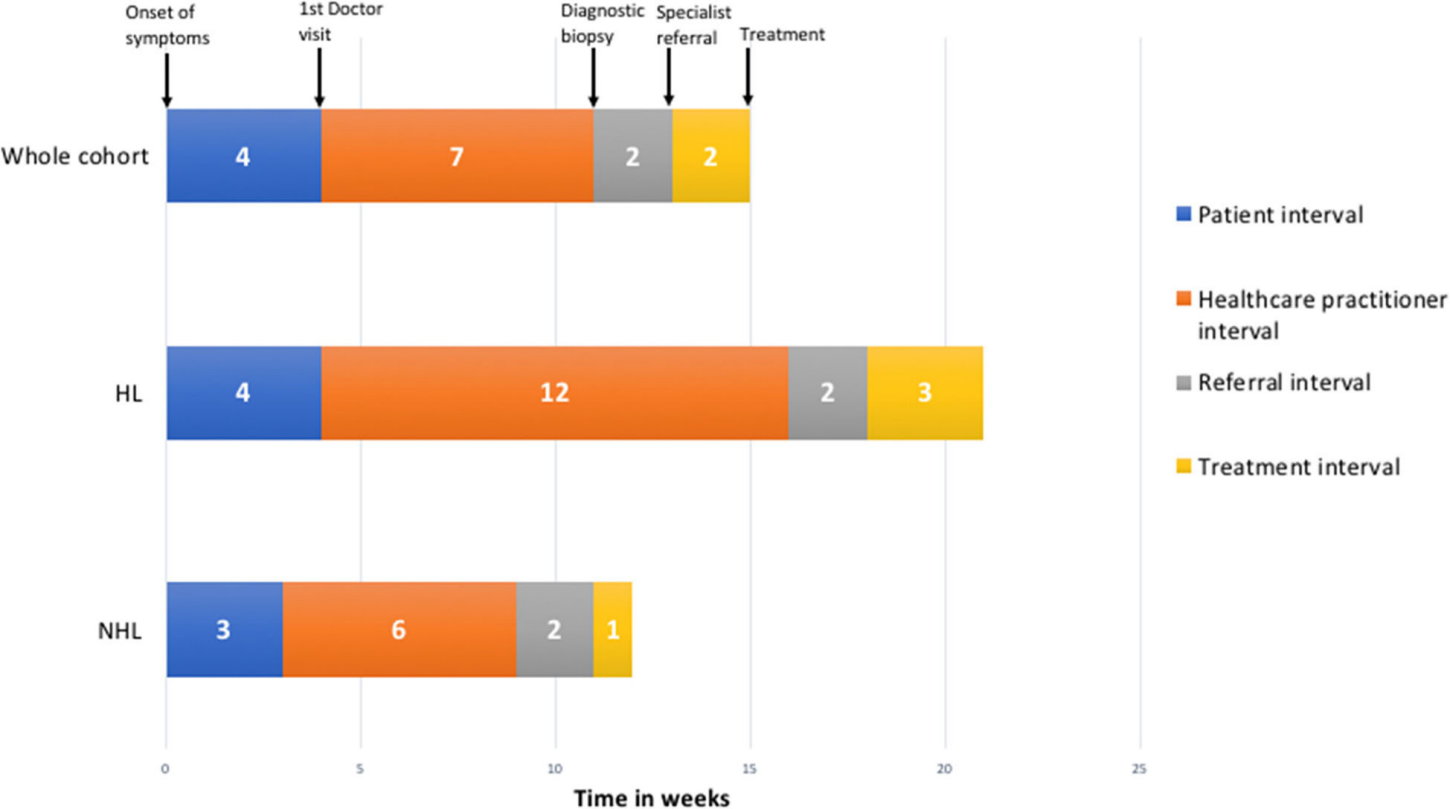
Median time to lymphoma diagnosis and treatment

The *patient interval* median was 4 weeks (*n* = 128 IQR 1.4–8.7), the *healthcare practitioner interval* median was 7 weeks (*n* = 133, IQR 3–16.5), the *referral interval* a median of 2 weeks (*n* = 152 IQR 1–3), and the *treatment interval* median of 2 weeks (*n* = 156 IQR 0.9–3). In 7% of patients (*n* = 11) the diagnosis of lymphoma was strongly suspected clinically and the patient was referred to the haematology / oncology service before a diagnostic biopsy, these patients were not included for assessment of *referral interval*. Table 2 summarises the pathway to diagnosis in weeks by time intervals described above. Figure 2 is a visual depiction of the overall time to pathway to diagnosis.

**Table 2:** Median and mean patient, diagnostic, referral and treatment time intervals

	Patient interval (wks)			Healthcare practitioner interval (wks)			Referral interval (wks)			Treatment interval (wks)		
	n	Median	p	n	Median	p	n	Median	p	n	Median	p
Sex												
Female	57	2	0.14	60	8	0.85	65	2	0.56	65	2	0.97
Male	71	4		73	7		87	2		91	1	
Age												
< 50 yr	75	4	0.59	79	6	0.26	88	2	0.54	88	2	0.27
≧ 50	53	3		54	7		64	2		68	1	
HIV												
Negative	91	4	0.41	93	7	0.58	105	2	0.20	112	1	0.29
Positive	37	2		40	8		47	2		44	2	
Lymphoma type												
NHL	96	3	0.15	99	6	0.01	112	2	0.64	117	1	< 0.01
HL	32	4		34	12		40	2		39	3	
Performance score												
ECOG 0	17	4	0.43	17	6	0.43	18	3	0.52	18	2	0.01
ECOG 1	57	4		59	8		71	2		73	2	
ECOG 2	19	2		21	9		23	2		27	1	
ECOG 3	25	2		25	7		29	2		28	1	
ECOG 4	10	2		11	5		11	2		10	2	
FNAC												
Not performed	76	3	0.38	78	5	0.04	91	2	0.86	94	1	0.08
Performed	52	4		55	9		61	2		62	2	
Stage												
Early-stage	45	3	0.66	47	4	0.02	56	2	0.12	58	2	0.16
Late-stage	83	4		86	9		96	2		98	1	
Clinical characteristics												
No peripheral lymph nodes	42	2	0.03	43	6	0.24	52	2	0.13	57	1	0.02
Peripheral lymph nodes	86	4		90	7		100	2		99	2	
No B symptoms	57	3	0.17	58	4	0.08	64	2	0.22	66	2	0.46
B Symptoms present	71	4		75	9		88	2		90	2	
Not on TB therapy	115	4	0.82	118	6	0.28	136	2	0.72	141	2	0.45
On TB therapy at diagnosis	13	3		15	10		16	2		15	2	
iagnosis made on lymph node	120	4	0.02	125	7	0.75	144	2	0.10	147	1	0.44
Diagnosis made on bone marrow	8	1		8	9		8	1		9	2

When the types of diagnostic delay are broken down by subtype of lymphoma there was a statistically significant longer median *healthcare practitioner interval* in patients with HL, (12 for HL vs 6 weeks for NHL, *p* < 0.01), when patients underwent an FNAC (median 9 vs 5 weeks, *p* = 0.04), and in patients with late-stage disease (median 9 vs 4 weeks, *p* = 0.02). Patients on TB therapy had non-statistically significant 4 week longer median health practitioner interval (median 10 vs 6 weeks, *p* = 0.28).

### Factors affecting diagnostic delay

Seventy of 133 (53%) patients for whom healthcare practitioner interval was known, had a diagnostic delay of > 6 weeks (*n* = 70). In univariate analysis, late-stage disease (*p* = 0.02) and HL (*p* < 0.01) were predictors for diagnostic delay. HIV status, age, race, performance status, emergency chemotherapy, presence of bone marrow involvement and TB treatment did not predict for diagnostic delay on univariate analysis. In multivariable analysis, patients with HL and late-stage disease continued to predict for diagnostic delay. Table 3 demonstrates the associations between the clinical covariates and diagnostic delay in multivariable analysis.

**Table 3:** Factors associated with diagnostic delay in multivariable logistic regression

	OR (95% CI)	*P*-value
FNAC performed (vs not performed)	1.4 (0.7–3.1)	0.38
Late-stage (vs early-stage)	2.3 (1.1–5.2)	0.04
HL (vs NHL)	3.0 (1.1–8.0)	0.03
Emergency chemotherapy (vs non-emergency)	0.6 (0.2–1.5)	0.24
HIV positive (vs negative)	0.9 (0.3–2.2)	0.76
Age ≥ 50 yr. (vs < 50 yr)	1.6 (0.7–4.0)	0.30

All the variables in the model are given above

*FNAC* Fine-needle aspiration and cytology, *HL* Hodgkin lymphoma, *NHL* Non-Hodgkin lymphoma

### Survival

**Fig. 3:**
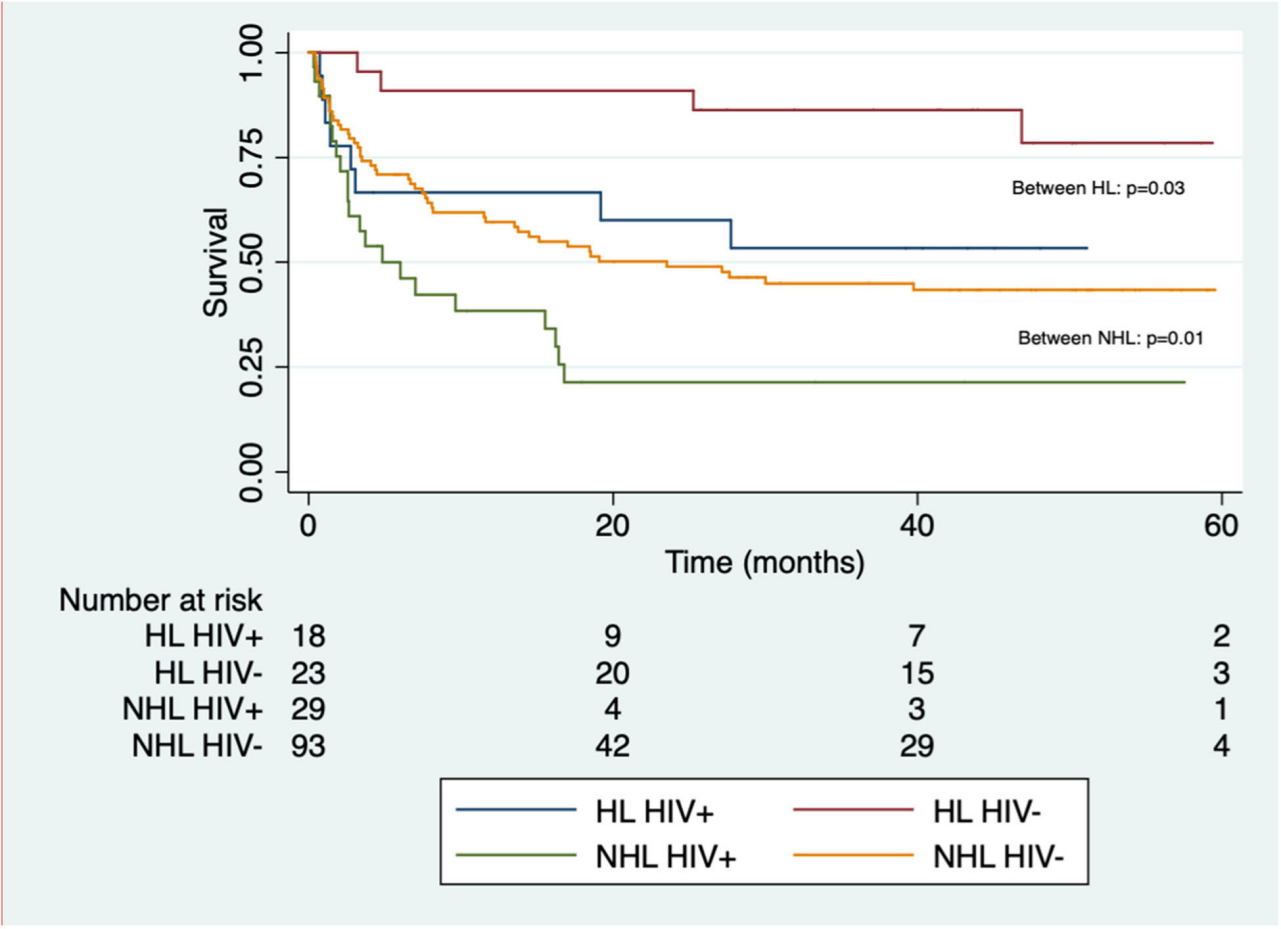
OS by type of lymphoma and HIV status

The 5-year OS was 46% (median survival time 27 months), based on total patient follow-up time of 3955 months, median 17 months (IQR 3–45 months). Two-year OS was 52%. The Kaplan-Meier curve for OS, stratified by type of lymphoma and HIV status shows a statistically significant difference in OS between HIV positive and negative patients in both HL and NHL (Fig. 3). In multivariable analysis, independent prognostic factors for OS were HIV infection, age > 50, poor performance status, late-stage disease and NHL. These factors predicted an increased risk for death both at 5 years and at 2 years (Table 4).

**Table 4:** Predictors (with hazard ratios (HR)) of Overall Survival (OS) and 2-year survival by multivariable Cox regression analysis

	OS HR (95% CI)	*P*-value	2 yr-survival HR (95% CI)	*P*-value
Late-stage (vs early-stage)	2.0 (1.1–3.8)	0.03	2.0 (1.2–3.4)	< 0.01
NHL (vs HL)	2.5 (1.1–5.0)	0.05	3.1 (1.5–6.3)	0.05
Emergency chemotherapy (vs non-emergency)	1.2 (0.6–2.3)	0.70	1.7 (0.9–2.9)	0.12
HIV positive (vs negative)	4.2 (1.9–9.4)	< 0.01	5.0 (2.0–12.4)	0.01
Performance status (vs ECOG = 0)				
ECOG 1	1.8 (0.5–6.3)	0.35	1.3 (0.5–3.1)	0.73
ECOG 2	3.7 (1.0–14.6)	0.06	2.5 (0.9–7.1)	0.09
ECOG 3	7.6 (2.1–27.4)	< 0.01	6.8 (2.6–17.8)	< 0.01
ECOG 4	4.5 (1.1–17.6)	0.03	6.2 (2.0–18.7)	< 0.01
Diagnostic delay	0.6 (0.3–1.0)	0.06	0.8 (0.5–1.3)	0.34
Age ≥ 50 (vs < 50)	4.1 (1.9–8.9)	< 0.01	1.7 (1.0–2.6)	0.03

All the variables in the model are given above

*HL* Hodgkin lymphoma, *NHL* Non-Hodgkin lymphoma, *ECOG* Eastern European Oncology Group

## Discussion

To the authors knowledge this is the first study to outline and quantify time intervals in the pathway to diagnosis of lymphoma in a high burden of HIV and TB setting. We examined factors that influence the length of time spent in these intervals. Key factors associated with a longer *healthcare practitioner interval* were HL (vs NHL), having an FNAC, and empiric TB therapy (although this did not reach statistical significance). A longer *healthcare practitioner interval* was also associated with late-stage disease, this correlation highlights that patients who have a longer diagnostic time will be more likely to have disease progression. Late-stage disease is associated with poorer outcomes to treatment and is included in prognostic scores both for HL and NHL. PLHIV did not experience longer delay to diagnosis, despite the risk of symptoms of lymphoma being more likely to be misattributed to HIV.

**Fig. 4:**
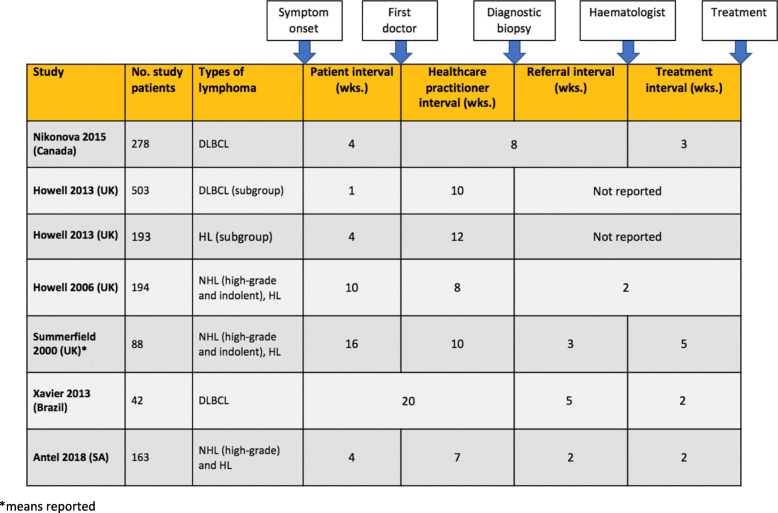
Existing literature on the time to diagnosis and treatment of lymphoma

A comparison with other studies is difficult due to differences in types of lymphoma included (especially if indolent lymphoma was included) and differences in definitions of the time intervals used in relation to different health systems. In order to conduct a meaningful comparison to the existing literature, we show the time intervals from studies looking at the time to diagnosis and treatment of lymphoma within the framework used in each study compared to our study (Fig. 4). Medians are shown except in the case of one study which only presented means [20]. The time intervals in our study are not dissimilar to that described in studies from high-income countries.

The term *healthcare practitioner interval* was specifically chosen rather than ‘diagnostic interval’ used in other studies because it highlights the active responsibility of the physician in this interval. There are three key physician factors for a patient to progress quickly through this interval: the ability to detect clinical features suggestive of lymphoma, a high suspicion for lymphoma, and access to a referral pathway for lymphadenopathy or to a diagnostic biopsy. During this interval the patient health-seeking behaviour plays a more minor role but (especially in the context of lymphoma without peripheral lymphadenopathy) patients may need to re-evaluate symptoms and seek help again. In this study, a very low proportion of patients (7%) were referred to haematology prior to a tissue diagnosis. This highlights firstly that lymphoma may not have been suspected and secondly that the role of the haematologist to facilitate making a diagnosis may not be recognised by primary care doctors. Within the health care practitioner interval, two key practices that thwart the diagnosis of lymphoma appear to be FNAC (especially repeated) and having an incorrect diagnosis of presumed EPTB.

The high proportion of repeat FNACs performed in this group of patients suggests physicians have an exaggerated diagnostic expectation of the utility of FNAC. There are numerous papers looking at the utility of the FNAC in the diagnosis of lymphoma with wide variations in reported accuracy. A recent meta-analysis reports a “median actionable diagnosis” of 74% for FNAC without ancillary methods but it is important to note that the meta-analysis did not attempt to address diagnostic accuracy (the diagnosis made on FNAC was taken as the diagnosis) [22]. The single paper in this meta-analysis that included centralised review of specimens submitted for a clinical trial (i.e. the FNA and excisional biopsy results were independently correlated), and that reflected community practice in that the FNACs were performed by different pathologists at different centres, reported only 12% diagnostic accuracy of the FNAC for lymphoma (and 29% when immunophenotyping was included) [8]. This finding is similar to our finding of diagnostic accuracy of 11%.

The high proportion of PLHIV on empiric TB therapy (26%) points to the relative ease with which physicians prescribe TB therapy in PLHIV with peripheral lymphadenopathy and constitutional symptoms. Being on TB therapy each added a median of 4 weeks to the time to diagnosis of lymphoma and is likely representative of a much larger group of patients who are misdiagnosed with TB and are never correctly diagnosed.

Patients with HL made up a small but important group of patients in this study: they were more likely to be misdiagnosed as TB, and were more likely to have a delay in diagnosis. This could be due to a higher frequency of B symptoms in HL, and a younger median age in whom a cancer diagnosis is less likely to be suspected.

We could not show an association between diagnostic delay and overall survival. This is consistent with other studies and likely relates to a heterogeneity in type and subtype of lymphoma (even within one lymphoma group like DLBCL the subtypes are heterogenous in response to standard therapy and rapidity of progression). Less aggressive subtypes may therefore take longer to present for healthcare but also have improved OS. However, the OS in this cohort was lower than expected when compared with the literature on NHL and HL, including in PLHIV. In PLHIV with HL, the 5-year OS in our cohort was only 53%, compared with 5-year OS of 88% from a highly-developed setting despite using the same treatment regimen of ABVD (adriamycin, doxorubicin, vinblastine, dacarbazine) [23], and a similar high proportion of patients having late-stage HL. The poorer outcome of the HL patients may be due to patients in this study having more advanced HIV and being less likely to be on ART therapy. Patients with diffuse large B-cell lymphoma (DLBCL) made up the large majority of patients with NHL and were treated with CHOP (cyclophosphamide, doxorubicin, vincristine and prednisone) or a CHOP-like regimen. Resource constraints meant that in this study cohort rituximab was used selectively (as per the provincial guidelines rituximab was used only for younger HIV negative patients with DLBCL). This will have adversely affected survival in the HIV positive patients with DLBCL since rituximab has shown benefit in HIV DLBCL with similar outcomes in HIV-negative DLBCL treated with R-CHOP [24].

Our study has limitations. The small study sample, especially HL would have affected results. The proportion of patients on TB therapy in our study is lower than has been reported and affected our ability to fully explore the magnitude of effect of TB therapy on diagnostic delay. Recall bias could have affected accurate reporting of symptoms and health-seeking behaviour. We sought to minimise this with the use of a single interviewer and a standard set of questions, but the insidious onset of symptoms in lymphoma makes this especially challenging. We also note that there were three different methods for data collection: telephonic interview, in-person interview and data collected from hospital files and there was no triangulation of data performed and that in some patients information regarding some of the intervals were missing. Interviewer bias may also have affected outcomes, the interviewer was not blinded to the patient’s HIV status or type of lymphoma. We therefore recognise that the ‘patient interval’ especially does need to be interpreted with caution. The other time intervals would have been less subject to these sources of bias.

When interpreting these results it is also important to recognise that there is likely significant under-diagnosis and under-reporting of cases of lymphoma in SSA. The estimated age-adjusted incidence rate for lymphoma in South Africa is 4.6 per 100,000/y [25] and despite the high incidence of HIV haematological malignancies are estimated to account for only 6% of new cancer cases in South Africa [26]. A significant proportion of patients with lymphoma in SSA may never reach a diagnosis of lymphoma and death may be misattributed to HIV or TB.

## Conclusions

With 163 patients, this is the largest study to report on time-to-diagnosis of lymphoma from SSA, and the largest study relating to HIV lymphoma diagnosis. We have ascertained that the longest delay in lymphoma diagnosis in our setting occurs in the diagnostic interval, termed the healthcare practitioner interval. The barriers to the diagnosis of lymphoma at the healthcare practitioner interval in a TB endemic area likely include: a high index of suspicion for TB obscuring the diagnosis of lymphoma, falsely high diagnostic perceptions of the utility of the FNAC, and a delay in accessing a lymph node biopsy. FNAC is not helpful nor cost-effective in the setting of suspected lymphoma and in addition, leads to diagnostic delay. Diagnostic delay is associated with advanced disease at presentation which is associated with higher mortality.

Future research is needed into the attitudes and beliefs of both lay people and healthcare practitioners about the significance of lymphadenopathy and barriers to obtaining a diagnostic biopsy. A best-practice algorithm for the investigation of lymphadenopathy in a TB endemic area, including implementation of the newer TB molecular diagnostic tests on lymph node tissue and a clear referral pathway for biopsy is needed to improve lymphoma diagnosis.

## Additional file

Additional file 1: Supplementary data (full dataset with identifiers removed). (XLSX 56 kb)

## Abbreviations

ART: Antiretroviral therapy
CI: Confidence Interval
EPTB: Extrapulmonary tuberculosis
FNAC: Fine-Needle Aspiration and Cytology
HL: Hodgkin Lymphoma
NHL: Non-Hodgkin lymphoma
OR: Odds Ratio
OS: Overall Survival
PLHIV: People Living with HIV
SSA: Sub-Saharan Africa
TB: Tuberculosis
